# Effect of Annealing Temperature on Mechanical Properties and Work Hardening of Nickel-Saving Stainless Steel

**DOI:** 10.3390/ma16113988

**Published:** 2023-05-26

**Authors:** Wei Pei, Shaoguang Yang, Kuo Cao, Aimin Zhao

**Affiliations:** Collaborative Innovation Center of Steel Technology, University of Science and Technology Beijing, Beijing 100080, China; 376744761@163.com (W.P.);

**Keywords:** nickel-saving stainless steel, ε-martensite transformation, twinning, dislocation density

## Abstract

Compared to Cr-Ni stainless steel, nickel-saving stainless steel is a low-cost austenitic stainless steel. We studied the deformation mechanism of stainless steel at various annealing temperatures (850 °C, 950 °C, and 1050 °C). The grain size of the specimen increases with increasing annealing temperature while the yield strength decreases, which follows the Hall–Petch equation. When plastic deformation occurs, dislocation increases. However, the deformation mechanisms can vary between different specimens. Stainless steel with smaller grains is more likely to transform into martensite when deformed. While twinning occurs when the grains are more prominent, the deformation results in twinning. The phase transformation during plastic deformation relies on the shear, so the orientation of the grains is relevant before and after plastic deformation.

## 1. Introduction

Nickel-saving austenitic stainless steel is a sub-stable austenitic stainless steel. It is formed by adding Mn, N, and other elements to the traditional austenitic stainless steel to partially or wholly replace the Ni element. Nickel-saving austenitic stainless steel shows excellent machinability, comprehensive mechanical properties, corrosion resistance, and decorative properties under the premise of reducing costs and saving noble metal resources. It is used in various industries, including architectural decoration, kitchenware, sanitary equipment, transportation equipment or parts, and more.

The nickel is essential to the resistance of localized corrosion in stainless steel. Replacing nickel with other elements, especially manganese (which forms sulfides), dramatically reduces the resistance of austenitic stainless steel to pitting corrosion. An effective way is to add nitrogen to austenitic stainless steel, which can improve the stability of austenite organization in austenitic stainless steel while improving resistance to pitting. Manganese increases the solubility of nitrogen in stainless steel and improves the austenitic stability of stainless steel, so it is not just a detriment in nickel-saving stainless steel [1,2].

The evolution of the microstructure of austenitic steels during deformation is also significant, as it affects the work hardening properties of the material. Austenite is usually in a sub-stable state at room temperature, and phase transformation occurs during deformation. For example, traditional Cr-Ni stainless steels, such as 304, will transform into α′-martensite during deformation, generating work hardening. In addition, some other high manganese steels do not form martensite during deformation, but they instead form twins or microbands, which can also obtain similar work hardening properties. Typically, they possess higher levels of carbon and manganese, which significantly improve the stability of austenite. These mechanisms of hardening during plastic deformation are called transformation-induced plasticity (TRIP) [3,4] and twinning-induced plasticity (TWIP) [5,6,7,8,9,10,11]. Both mechanisms positively affect the homogeneous deformation of plastic deformation and, consequently, increase the overall elongation of austenitic steels [12,13,14].

In addition to the stability of austenite, the stacking fault energy (SFE) has been proposed [15,16,17,18,19]. It supposes that the SFE is the Gibbs free energy required to create a platelet of ε-martensite of a thickness of only two atomic layers. With the increase in SFE, the deformation mechanism of high manganese steel from TRIP becomes TWIP and then becomes dislocation planner slip. Recently, researchers have studied the SFE in regard to on temperature and chemical composition. It has a significant influence on the mechanical properties of alloys. In contrast to high manganese TWIP steels, more chromium and less carbon in stainless steel lead to a lower SFE [18,20,21], which makes the martensitic transformation much easier during plastic deformation.

SFE increases with increasing temperature [8,22]. Moreover, the effective SFE also depends on the crystallographic orientation [23], which can be understood by the value of the Schmid factor when slipping [24]. Grades 304 L and 316 L stainless steel will transform to ε-martensite during the drawing process, followed by the formation of α′ martensite near the ε-martensite [25,26]. During cold working, the martensite content in AISI 301 increases when the deformation is high or the deformation temperature is low [27]. Moreover, increasing the strain rate and the ASTM austenite grain size number reduces the amount of strain-induced martensite [28]. This study aims to understand the recrystallization during heat treatment after the cold roll and the strain-hardening behavior during plastic deformation. The fascinating connection between the stress–strain curve and dislocation density has been thoroughly investigated. A summary of strain-hardening characteristics and micro-structure transformation is also presented.

## 2. Materials and Method

The stainless steel that saves nickel was melted in an induction furnace. Its chemical composition included 16.61% manganese, 11.95% chromium, 0.876% nickel, 0.624% copper, 0.13% carbon, 0.19% nitrogen, and a balanced weight percentage of iron to billets of 80 mm × 30 mm. It was then air cooled to room temperature. The start and finish forging temperatures were controlled to be higher than 1150 °C and 900 °C, respectively. After being held at 1100 °C for 1 h to obtain a homogeneous microstructure, the billet was hot rolled onto a plate with a thickness of 5 mm. An approximately 65% single pass reduction was applied, and the sheet was cold rolled to a thickness of 1.7 mm. Samples were obtained from cold-rolled plates and heat-treated to observe the metallographic structure. The specimens were heated to 850 °C, 950 °C, and 1050 °C for 15 min, and then were quenched in water to room temperature. The steels that were annealed at three temperatures are named S850, S950 and S1050. The size and orientation of grains were observed by electron backscatter diffraction (EBSD). The tensile strength was evaluated by obtaining deformed specimens at room temperature using a universal testing machine identified by CMT4105. Tensile samples made of annealed steel, with a length of 25 mm, were tested at a rate of 1 mm/min. The specimens deformed with strains of 0.1, 0.2, 0.3, and 0.4 were analyzed using X-ray diffraction (XRD) and EBSD. Before the tests, electrochemical polishing was used to eliminate surface stress. The polished fluid was a 10% perchloric acid alcohol solution, and the current density during the polishing was 5 A/cm^−2^. The Bruker D8 Advance X-ray diffractometer was used to collect X-ray diffraction patterns with a Cu-Kα radiation source. Each run was conducted in the range of 40° to 120°. EBSD measurements were carried out on the JSM 7200F equipped with an EDAX Velocity Super attachment. 

## 3. Result

### 3.1. Microstructure and Mechanical Properties

**Figure 1:**
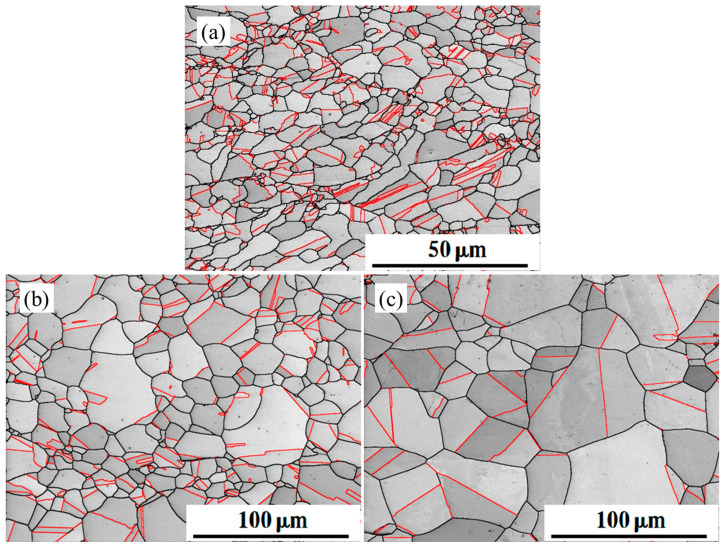
The EBSD band contrast maps of sample after annealing at (**a**) 850 °C, (**b**) 950 °C, and (**c**) 1050 °C. The red lines represent twin boundaries and the black lines represent grain boundaries.

EBSD was used to characterize the microstructure of experimental steels after recrystallization annealing at 850 °C, 950 °C, and 1050 °C for 15 min. The EBSD band contrast maps in Figure 1 indicate grain coarsening with increasing annealing temperature. Many annealed twins were found in the annealed specimens, and the grain sizes of the three samples were 2.3 μm, 6.4 μm, and 15.5 μm, respectively.

The grains still appeared as stretched elongated strips in S850, and many twins were parallel to the elongation direction. When the annealing was increased to 950 °C, the length of the grains in each direction tended to be uniform. The twins were also annexed to each other during recrystallization to form structures of more excellent dimensions. One part of the twin crossed the grains, while the other part was inserted into the grain from the grain boundaries. Once the annealing temperature reached 1050 °C, the width of the twins continued to widen as the grains grew. The number of twin boundaries was reduced due to mutual annexation between twins.

**Figure 2:**
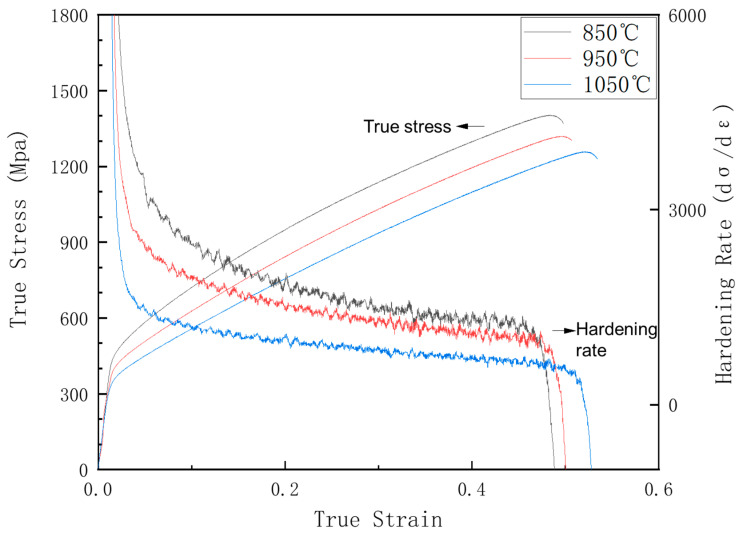
The true stress–true strain curves for S850, S950, and S1050.

The true stress–true strain curves for various annealing conditions are shown in Figure 2. Strength decreases with increasing annealing temperature for the increased grain size. The yield strengths of S850, S950, and S1050 were 418 MPa, 354 MPa, and 316 MPa, respectively, while the ultimate tensile strength was 1403 MPa, 1320 MPa, and 1258 MPa, respectively. However, the elongation was not significantly increased by the increased annealing temperature, which was 0.48, 0.50, and 0.52 at the three annealed temperatures. Similarly, the work hardening rate decreased with increasing annealing temperature. The work hardening rate gradually decreased during the deformation, and no reversion occurred.

### 3.2. XRD Test and Dislocation Density

**Figure 3:**
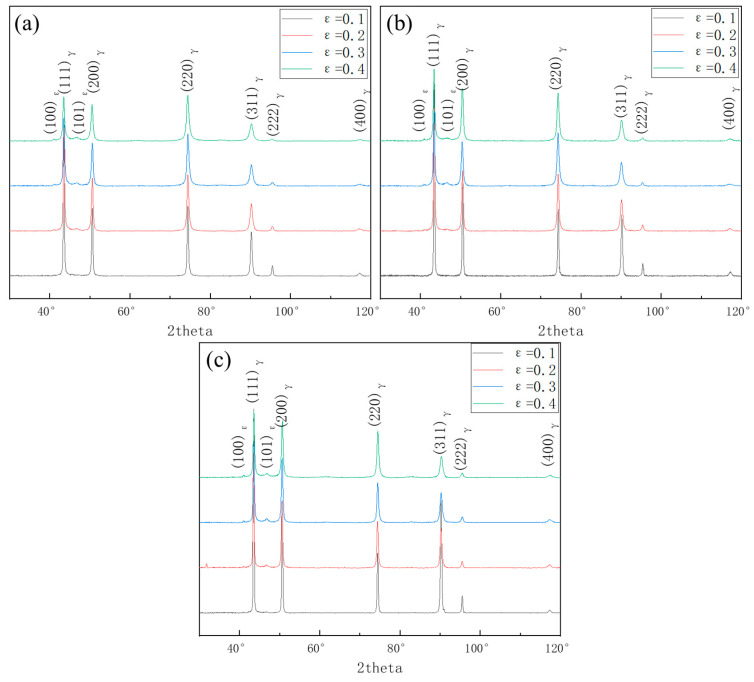
XRD diffraction patterns of deformed (**a**) S850, (**b**) S950, and (**c**) S1050.

Specimens after annealing at different temperatures were deformed to different rates of deformation. The XRD tests were carried out on the specimens, and the curve is shown in Figure 3. Austenite was still the primary phase in stainless steel after it deformed. ε-martensite is also a deformed martensite phase, but almost no α′-martensite was observed. However, it was not easy to obtain the relevant phase ratios from the XRD results for the overlap of diffraction peck of austenite and ε-martensite.

**Figure 4:**
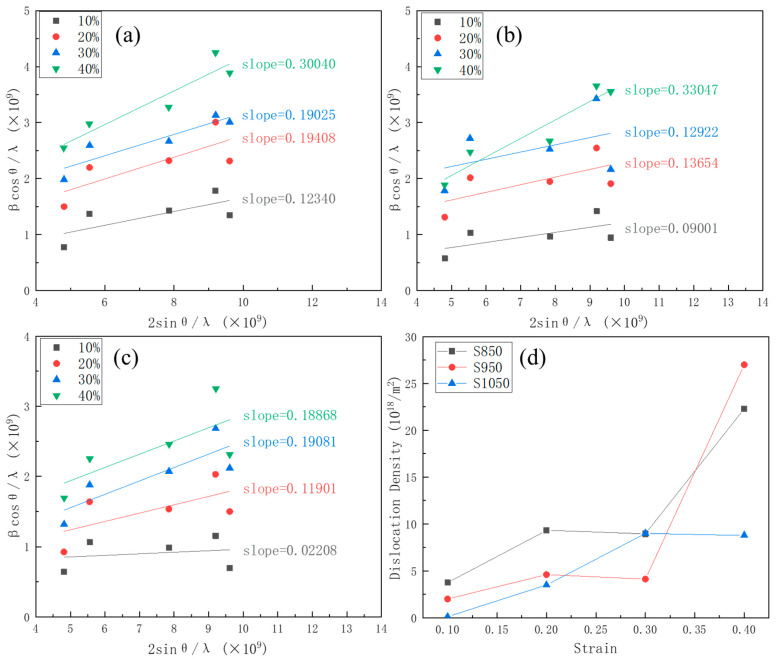
(**a**–**c**) Linear fitting based on the Williamson–Hall equation and (**d**) the density of dislocation at different deformation rate.

Not only do phase transitions occur during deformation, but dislocation also accrues. The increasing density of dislocations can describe this process. The dislocation density can be calculated from the peak position and full width at half maxima (FWHM) through the use of the Williamson–Hall equation.

(1) $$\frac{\beta cos\theta }{\lambda }=\frac{0.9}{D}+2\varepsilon \frac{\sin\theta }{\lambda }$$

(2) $$\rho =16.1{\left(\frac{\varepsilon }{b}\right)}^{2}$$

where *θ* is the diffraction angle of the peak, β is the FWHM of the peak, *λ* is the wavelength of the incident X-ray beam (0.15406 nm), *D* is the crystallite size, *ε* is a heterogeneous strain, and *b* is the magnitude of the vector (0.2552 nm in the present material), respectively. The results of the dislocation density are shown in Figure 4. 

The dislocation density of stainless steel is inversely proportional to the annealing temperature in the early stages of deformation. The increased annealing temperature contributes to the disappearance of dislocations during recrystallisation. A noticeable shift in dislocation density occurs in the deformation. The trend in dislocation density during the deformation of S850 and S950 is about the same, with the change not being substantial until the strain is 0.3. However, a dramatic increase in dislocation density with the strain occurred to 0.4; the dislocation density increased from 8.95 × 10^18^ m^−2^ to 22.30 × 10^18^ m^−2^ and from 4.13 × 10^18^ m^−2^ to 26.99 × 10^18^ m^−2^, respectively. In contrast, the experimental steel annealed at 1050 °C showed a gradual increase in the value of dislocation density until the strain was raised to 0.3. The dislocation density, however, did not changed significantly when the strain was increased.

### 3.3. EBSD Test and Phase Transitions

**Figure 5:**
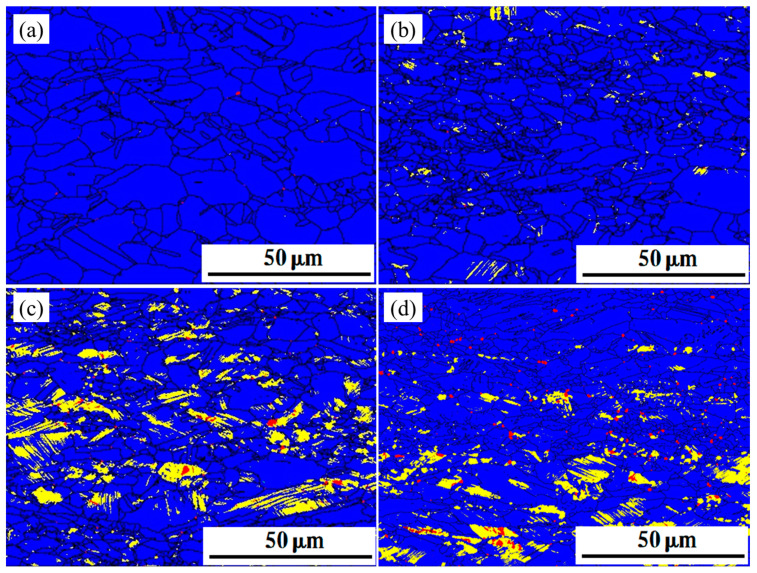
The EBSD phase maps of deformed S850; blue part for austenite, yellow part for ε-martensite and red part for α′-martensite. (**a**) ε = 0.1 (**b**) ε = 0.2 (**c**) ε = 0.3 (**d**) ε = 0.4.

**Figure 6:**
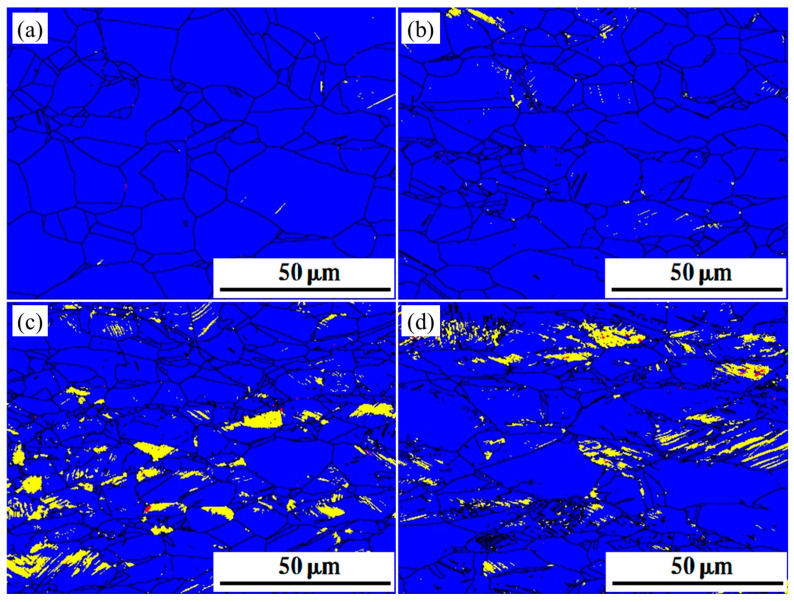
The EBSD phase maps of deformed S950; blue part for austenite, yellow part for ε-martensite and red part for α′-martensite. (**a**) ε = 0.1 (**b**) ε = 0.2 (**c**) ε = 0.3 (**d**) ε = 0.4.

**Figure 7:**
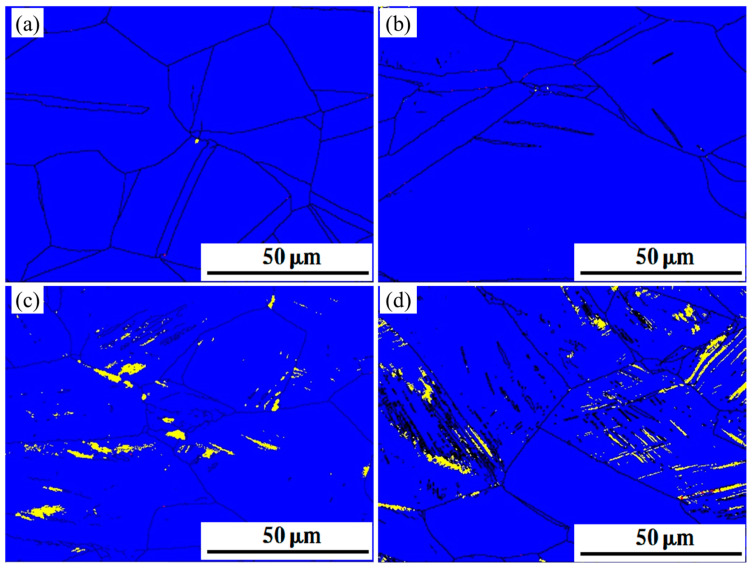
The EBSD phase maps of deformed S1050; blue part for austenite, yellow part for ε-martensite and red part for α′-martensite. (**a**) ε = 0.1 (**b**) ε = 0.2 (**c**) ε = 0.3 (**d**) ε = 0.4.

Specimens with different rates of deformation were tested in EBSD. The phase maps of the deformed specimens are shown in Figure 5, Figure 6 and Figure 7. Austenite (blue in EBSD phase maps) remained the dominant phase in deformed specimens. ε-martensite (yellow in EBSD phase maps) was detected in all three specimens after deformation, and the content of ε-martensite increased with the deformation rate. The proportion of ε-martensite was lower in steels annealed at higher temperatures, with maximum ε-martensite content after deformation of 12.3%, 6.92%, and 2.1% for S850, S950, and S1050, respectively. Two types of ε-martensite were present in the deformed steel. A lamellar morphology formed within the larger grains, while blocky morphology formed within the smaller grains. In addition, α′-martensite (red in EBSD phase maps) was observed inside the deformed S850 and S950. α′-martensite does not appear in isolation but within or along the edges of ε-martensite. Both annealing temperature and deformation affect the α′-martensitic transformation, as does the ε-martensitic transformation. The α-martensitic transformation is also suppressed once the annealing temperature is increased so that α′-martensite is almost invisible in deformed S1050, in which twins were observed parallel to ε-martensite.

## 4. Discussion

The stainless steel, after cold rolling, accumulates a large amount of distortion energy within the deformed matrix. During recrystallization, newly formed grains are created along the original grain boundaries and grow by utilizing the present distortion energy. Crystals are prone to recrystallization and grow after grain nucleation during annealing. In addition, the formation of twins can reduce interfacial energy because twin boundaries have lower interfacial energy than ordinary grain boundaries. The formation of annealing twins could decrease grain growth’s boundary energy and increase grain boundary mobility [29,30]. The newly formed grains will nucleate on the grain boundaries, but there will also be lattice defects such as stack faults and dislocation tangles inside the deformed grains, providing nucleation points for newly formed grains. Newly formed grains at grain boundaries usually have random orientations, so twins are more likely to form inside the original grains [31]. 

More grains are nucleated at the grain boundaries during annealing at a higher annealing temperature. The higher temperatures will also promote grain boundary migration, resulting in the new-born grains being swallowed up the deformed grain. After the annealing process at higher temperatures, there are fewer twin boundaries and more grains of larger size. If the annealing is at a lower temperature, the recrystallization nucleation rate decreases, resulting in slower grain growth. As a result, the grain sizes become smaller, and twins occur more frequently. Figure 1 shows an overall decrease in twin boundaries with increasing annealing temperature.

As an essential obstacle to dislocation slip, grain boundaries significantly influence the yield strength of stainless steel. The yield strength of steel is related to grain size, and the yield strength and grain size satisfy the Hall–Petch equation.

(3) $$\sigma_{y}=\sigma_{0}+kd^{-1/2}$$

where $\sigma_{y}$ is the yield strength, $\sigma_{0}$ is the intrinsic strength associated with the material, k is a constant, and d is the grain size (μm). According to mathematical fitting by grain size and yield strength, this experimental steel has the $\sigma_{0}$ of 253.4 MPa and k of 250.4 MPa·μm^1/2^.

**Figure 8:**
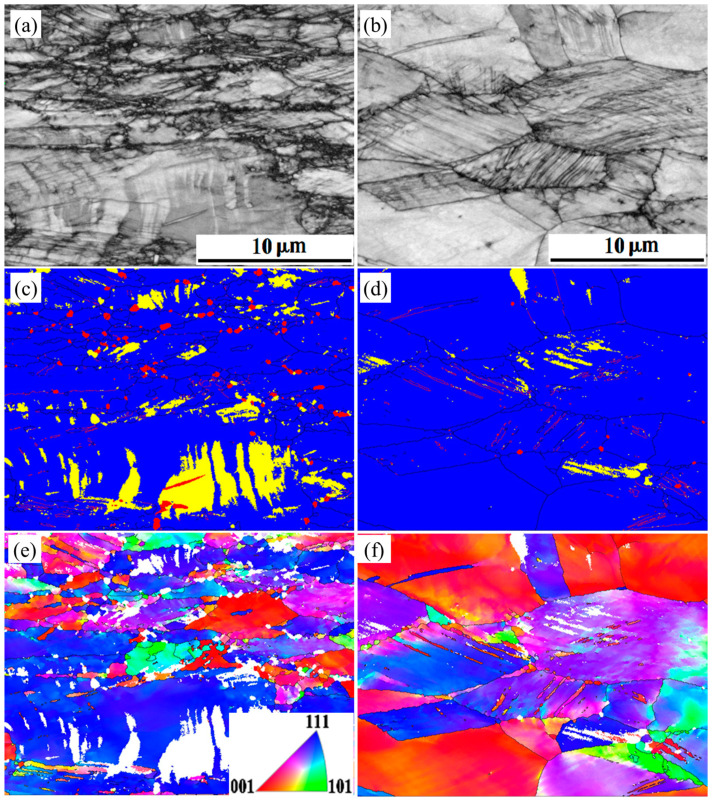
The EBSD result of S850 (**a**,**c**,**e**) and S950 (**b**,**d**,**f**) at a strain rate of 0.4. (**a**,**b**) The band contrast maps; (**c**,**d**) Phase maps (blue for austenite, yellow for ε-martensite and red for α′-martensite; the red lines for twin boundaries); (**e**,**f**) IPF maps of deformed austenite.

The present study assumes that the plastic deformation mechanism is determined by the annealing temperature. Increasing the annealing temperature would change the hardening mechanism from martensitic to twinning transformation. Deformation twinning is strongly inhibited in smaller grains [32,33]. A more significant critical shear stress is required to produce twinning within finer grains, making it challenging to form deformation twins during deformation. However, finer grains contain more grain boundaries, which can quickly generate stress concentration during elongation. Thus, multiple slips can quickly occur near grain boundaries, and this significant grain boundary reaction accelerates the martensitic transformation [34]. These factors ultimately result in a more significant fraction of martensite within the finer grains and a relatively small increase in dislocation density. In addition, the martensitic transformation during elongation is also related to the orientation of the crystal [35]. Specimens annealed at lower temperatures still retain the inhomogeneity of grain orientation after cold rolling [36]. They are more prone to martensitic transformation and to forming bulk martensite in grains. Therefore, martensitic transformation occurs as the primary work hardening mechanism in the early stages of deformation in S850 and S950. Moreover, when the proportion of martensite reaches its maximum, it acts as a barrier to dislocation slip, causing a rapid increase in dislocation density. In contrast, the grain size in S1050 is more prominent, making the critical shear stress required for twinning during deformation much lower so that twinning is no longer complex. However, the reduction in grain boundaries due to grain growth makes it difficult to accumulate the micro stresses required for martensitic transformation and inhibits the generation of martensite. The comprehensive causes result in the hardening mechanism that shifts from martensitic transformation to twinning transformation with increased annealing temperature, as shown in Figure 8.

The grains will rotate due to plastic deformation and form a deformation texture [37]. Grains whose <111> orientation or <100> crystal orientation is parallel to the direction of deformation will be preserved more after plastic deformation [34,38]. A correlation also exists between twinning and martensitic transformation with grain orientation [39], as shown in Figure 8. Twins and ε-martensite mostly appear inside the grains whose <111> direction is parallel to the deforming direction, while the grains whose <100> direction is parallel to the deforming direction do not. A darker color of ε-martensite in the BC map implies a higher dislocation density or micro-strain. In contrast, the α′-martensite region is lighter for consuming the dislocations accumulated during plastic deformation. The grain correlations such as <−1–11>γ||<02–21>ε are present in the deformed steel. Twins, rather than martensite, will appear in specimens with larger grains. Twins formed with grain orientations <100> parallel to the tensile direction will also be in a stable orientation, as Figure 8 shows.

## 5. Conclusions

In the present study, nickel-saving stainless steel, after cold rolling, was heat treated at different temperatures and then deformed. We have concluded the following:

(1) The yield strength of nickel-saving obeys the Hall–Petch equation, and the increase in grain size leads to a decrease in yield strength. The yield strengths of S850, S950, and S1050 were 418 MPa, 354 MPa, and 316 MPa, respectively.
(2) Through plastic deformation, dislocations were created in the stainless steel. Additionally, phase transformation or twinning was utilized to achieve work hardening behavior. As the annealing temperature increases, the work hardening mechanism transitions from martensitic transformation to twinning transformation.
(3) The grains rotate during deformation, and the grain orientation of austenite grains parallel to the deformation direction after deformation is either <111> or <100>. The generated ε-martensite and austenite will have an orientation relationship such as <−1–11>γ||<02–21>ε.

## Data Availability

This study did not report any data.

## References

[1] Pistorius P.C. Low-Nickel Austenitic Stainless Steels: Metallurgical Constraints. Proceedings of the Twelfth International Ferroalloys Congress.

[2] Aravindkumar D., Thirumalai R. (2021). Investigations on Microstructural Characteristics and Mechanical Properties of 316 L Stainless Steel Welded Joints Using Nickel Coated Filler Material by Gas Tungsten Arc Welding. Mater. Res. Express.

[3] Chattopadhyay S., Anand G., Chowdhury S.G., Manna I. (2018). Effect of Reverse Austenitic Transformation on Mechanical Property and Associated Texture Evolution in AISI 316 Austenitic Stainless Steel Processed by Low Temperature Rolling and Annealing. Mater. Sci. Eng. A.

[4] Yang X.-S., Sun S., Ruan H.-H., Shi S.-Q., Zhang T.-Y. (2017). Shear and Shuffling Accomplishing Polymorphic Fcc γ → Hcp ε → Bct α Martensitic Phase Transformation. Acta Mater..

[5] Gutierrez-Urrutia I., Zaefferer S., Raabe D. (2010). The Effect of Grain Size and Grain Orientation on Deformation Twinning in a Fe–22wt.% Mn–0.6wt.% C TWIP Steel. Mater. Sci. Eng. A.

[6] Gao Y.-J., Deng Q.-Q., Liu Z., Huang Z.-J., Li Y.-X., Luo Z.-R. (2020). Modes of Grain Growth and Mechanism of Dislocation Reaction under Applied Biaxial Strain: Atomistic and Continuum Modeling. J. Mater. Sci. Technol..

[7] Zhu Y.T., Liao X.Z., Wu X.L. (2012). Deformation Twinning in Nanocrystalline Materials. Prog. Mater. Sci..

[8] Ye T., Zhao F., Chen L., Jiang K., Deng Q., Chen Y., Wang Q., Suo T. (2021). Effect of Strain Rate and Low Temperature on Mechanical Behaviour and Microstructure Evolution in Twinning-Induced Plasticity Steel. Mater. Sci. Eng. A.

[9] Abbas M.A., Hong S.H., Yusupov D., Kang G.C., Choi J.-W., Jumaev E., Park H.J., Kim K.B. (2023). Evolution of Microstructure and Mechanical Properties of a Ti80(CoFeNi)20 Ultrafine Eutectic Composite during Thermal Processing. Intermetallics.

[10] Jumaev E., Abbas M.A., Mun S.C., Song G., Hong S.-J., Kim K.B. (2021). Nano-Scale Structural Evolution of Quaternary AlCrFeNi Based High Entropy Alloys by the Addition of Specific Minor Elements and Its Effect on Mechanical Characteristics. J. Alloys Compd..

[11] Jumaev E., Hong S.H., Kim J.T., Park H.J., Kim Y.S., Mun S.C., Park J.-Y., Song G., Lee J.K., Min B.H. (2019). Chemical Evolution-Induced Strengthening on AlCoCrNi Dual-Phase High-Entropy Alloy with High Specific Strength. J. Alloys Compd..

[12] Idrissi H., Renard K., Schryvers D., Jacques P.J. (2010). On the Relationship between the Twin Internal Structure and the Work-Hardening Rate of TWIP Steels. Scr. Mater..

[13] Zhenli M., Di T., Aimin Z., Haitao J. (2012). Mechanical Properties and Microstructure Evolution During Deformation of Fe–Mn–C TWIP Steel. Steel Res. Int..

[14] Xu D.M., Li G.Q., Wan X.L., Xiong R.L., Xu G., Wu K.M., Somani M.C., Misra R.D.K. (2017). Deformation Behavior of High Yield Strength—High Ductility Ultrafine-Grained 316LN Austenitic Stainless Steel. Mater. Sci. Eng. A.

[15] Curtze S., Kuokkala V.-T., Oikari A., Talonen J., Hänninen H. (2011). Thermodynamic Modeling of the Stacking Fault Energy of Austenitic Steels. Acta Mater..

[16] Lee T.-H., Shin E., Oh C.-S., Ha H.-Y., Kim S.-J. (2010). Correlation between Stacking Fault Energy and Deformation Microstructure in High-Interstitial-Alloyed Austenitic Steels. Acta Mater..

[17] Tian Y., Gorbatov O.I., Borgenstam A., Ruban A.V., Hedström P. (2017). Deformation Microstructure and Deformation-Induced Martensite in Austenitic Fe-Cr-Ni Alloys Depending on Stacking Fault Energy. Met. Mater. Trans. A.

[18] Talonen J., Hänninen H. (2007). Formation of Shear Bands and Strain-Induced Martensite during Plastic Deformation of Metastable Austenitic Stainless Steels. Acta Mater..

[19] Lu J., Hultman L., Holmström E., Antonsson K.H., Grehk M., Li W., Vitos L., Golpayegani A. (2016). Stacking Fault Energies in Austenitic Stainless Steels. Acta Mater..

[20] Das A. (2016). Revisiting Stacking Fault Energy of Steels. Met. Mater. Trans. A.

[21] Yonezawa T., Suzuki K., Ooki S., Hashimoto A. (2013). The Effect of Chemical Composition and Heat Treatment Conditions on Stacking Fault Energy for Fe-Cr-Ni Austenitic Stainless Steel. Met. Mater. Trans. A.

[22] Wittig J.E., Pozuelo M., Jiménez J.A., Frommeyer G. (2009). Temperature Dependent Deformation Mechanisms of a High Nitrogen-Manganese Austenitic Stainless Steel. Steel Res. Int..

[23] Karaman I., Sehitoglu H., Gall K., Chumlyakov Y.I., Maier H.J. (2000). Deformation of Single Crystal Hadfield Steel by Twinning and Slip. Acta Mater..

[24] Chen G., Rahimi R., Xu G., Biermann H., Mola J. (2020). Impact of Al Addition on Deformation Behavior of Fe–Cr–Ni–Mn–C Austenitic Stainless Steel. Mater. Sci. Eng. A.

[25] Spencer K., Véron M., Yu-Zhang K., Embury J.D. (2009). The Strain Induced Martensite Transformation in Austenitic Stainless Steels: Part 1—Influence of Temperature and Strain History. Mater. Sci. Technol..

[26] Das A., Sivaprasad S., Ghosh M., Chakraborti P.C., Tarafder S. (2008). Morphologies and Characteristics of Deformation Induced Martensite during Tensile Deformation of 304 LN Stainless Steel. Mater. Sci. Eng. A.

[27] Mirzadeh H., Najafizadeh A. (2008). Correlation between Processing Parameters and Strain-Induced Martensitic Transformation in Cold Worked AISI 301 Stainless Steel. Mater. Charact..

[28] Varma S.K., Kalyanam J., Murk L.E., Srinivas V. (1994). Effect of Grain Size on Deformation-Induced Martensite Formation in 304 and 316 Stainless Steels during Room Temperature Tensile Testing. J. Mater. Sci. Lett..

[29] Poelt P., Sommitsch C., Mitsche S., Walter M. (2006). Dynamic Recrystallization of NI-Base Alloys-Experimental Results and Comparisons with Simulations. Mater. Sci. Eng. A.

[30] Mandal S., Bhaduri A.K., Sarma V.S. (2012). Influence of State of Stress on Dynamic Recrystallization in a Titanium-Modified Austenitic Stainless Steel. Metall. Mater. Trans. A.

[31] Wang Y., Jia Z., Gao Z., Liu D. (2023). Continuous Dynamic Recrystallization Nucleation Mechanism and Annealing Twin Evolution with Respect to Grain Growth in a Nickel-Based Superalloy. J. Cent. South Univ..

[32] Hung C.-Y., Bai Y., Tsuji N., Murayama M. (2021). Grain Size Altering Yielding Mechanisms in Ultrafine Grained High-Mn Austenitic Steel: Advanced TEM Investigations. J. Mater. Sci. Technol..

[33] Lee S.-I., Lee S.-Y., Han J., Hwang B. (2019). Deformation Behavior and Tensile Properties of an Austenitic Fe-24Mn-4Cr-0.5C High-Manganese Steel: Effect of Grain Size. Mater. Sci. Eng. A.

[34] Ma D., Yang P., Gu X., Cui F. (2022). Influences of Initial Microstructures on Martensitic Transformation and Textures during Cold Rolling and Tensile Mechanical Properties in High Manganese TRIP Steel. Mater. Sci. Eng. A.

[35] Wang H., Sun X., Yang P., Mao W., Meng L. (2015). Analysis of the Transformation-Induced Plasticity Effect during the Dynamic Deformation of High-Manganese Steel. J. Mater. Sci. Technol..

[36] Nezakat M., Akhiani H., Sabet S.M., Szpunar J. (2017). Electron Backscatter and X-Ray Diffraction Studies on the Deformation and Annealing Textures of Austenitic Stainless Steel 310S. Mater. Charact..

[37] Sevsek S., Brasche F., Molodov D.A., Bleck W. (2019). On the Influence of Grain Size on the TWIP/TRIP-Effect and Texture Development in High-Manganese Steels. Mater. Sci. Eng. A.

[38] Pei W., Zhang Y., Yang S., Li X., Zhao A. (2022). Study of Work-Hardening Behavior of High Manganese Steel during Compression. Mater. Res. Express.

[39] Xie P., Shen S., Wu C., Chen J. (2021). Abnormal Orientation Relation between Fcc and Hcp Structures Revealed in a Deformed High Manganese Steel. J. Mater. Sci. Technol..

